# The association between adverse pregnancy outcomes and maternal human papillomavirus infection: a systematic review protocol

**DOI:** 10.1186/s13643-017-0443-5

**Published:** 2017-03-11

**Authors:** Joseph Niyibizi, Nadège Zanré, Marie-Hélène Mayrand, Helen Trottier

**Affiliations:** 1 Department of Social and Preventive Medicine, School of Public Health, Université de Montréal, Pavilion 7101, Avenue du Parc, Montreal, QC H3N 1X7 Canada; 2 Department of Social and Preventive Medicine, Sainte-Justine Hospital Research Center, Université de Montréal, 3175 Côte Sainte-Catherine, Room A-830, Montreal, H3T 1C5 QC Canada; 3 Department of Obstetrics and Gynecology, Research Centre of University of Montreal Hospital Centre (CRCHUM) and University of Montreal, Tour Saint-Antoine, 850, rue St-Denis, Montreal, QC H2X 0A9 Canada

**Keywords:** Pregnant women, Human papillomavirus, Adverse pregnancy outcomes, Systematic review, Protocol

## Abstract

**Background:**

Human papillomavirus (HPV) is the most prevalent genital infection, especially in young women of reproductive age. In vitro and animal model experiments provide compelling evidence of the harmful effect of HPV on pregnancy outcomes, but results from epidemiologic studies are inconclusive. We aim to determine the strength of the relationship between adverse pregnancy outcomes (APO) and HPV infection and assess its consistency across studies, by systematically reviewing the literature.

**Methods:**

The search strategy has been developed on the basis of the PICOS framework: Population (pregnant women); Exposure (HVP infection confirmed by HPV testing); Comparator (pregnant women without HPV infection); Outcomes (miscarriage, spontaneous preterm birth, low birth weight, preterm premature rupture of membranes, pregnancy-induced hypertensive disorders and intrauterine growth restriction) and Study design (observational studies). We will search three information sources: (1) electronic databases (MEDLINE, EMBASE, and EBM Reviews databases); (2) Grey literature (Google Scholar and Web of Science conference proceedings); and (3) citing and cited articles of included studies. Two reviewers (JN, NZ) will independently and in duplicate screen identified articles, select eligible studies, and extract data. Discrepancies will be resolved by consensus and otherwise by discussion with the other authors (MHM, HT). Quality of included studies will be assessed using the Effective Public Health Practice Project (EPHPP) Quality Assessment Tool for Quantitative Studies. We will narratively synthesize extracted data whether meta-analysis is conducted or not. Meta-analysis of each outcome will be performed, and where appropriate, an average measure of association will be computed. We will use the Grading of Recommendations Assessment, Development and Evaluation (GRADE) approach to assess and grade the strength of confidence in cumulative estimate.

**Discussion:**

Comprehensive and high-quality evidence of a negative effect of HPV on pregnancy outcomes might be an additional motivation for HPV vaccination. Absence of such relationship could dispel anxiety and reassure HPV-infected pregnant women and clinicians. Findings of a poor level of confidence will allow identification of current knowledge gaps on HPV-pregnancy outcome relationship that need further research.

**Systematic review registration:**

PROSPERO CRD42016033425

**Electronic supplementary material:**

The online version of this article (doi:10.1186/s13643-017-0443-5) contains supplementary material, which is available to authorized users.

## Background

Human papillomavirus (HPV) is the most common sexually transmitted infection in adults [1, 2]. The lifetime probability of genital HPV acquisition is estimated to be more than 80% in both women and men by the age of 45 years [2]. The highest incidence rates occur in young adults, just after the onset of sexual activity [3]. Longitudinal studies in young women 17–24 years of age-reported incidence rates ranging from 15.7 to 29.4 HPV infections of all types per 1000 women-months [4]. Cohort studies of women in their thirties showed a lower incidence rate, between 5.2 and 13.4 HPV infections any type per 1000 women-months [4].

Similarly, the prevalence is higher in younger than in older age groups. A multi-country meta-analysis estimated the global HPV prevalence at 11.7% (confidence interval 95% 11.6%-11.7%) in women with normal cytology with an important variation within and between geographic regions [5]. The prevalence peaks at around 25 years and decreases thereafter. In some populations, a smaller second peak is observed at 45 years [5, 6].

In men, the prevalence estimates of genital HPV infection vary widely across studies ranging from 1 to 84% [7]. Unlike in women, the HPV prevalence curve in men peaks at older ages and remains steady high or decreases slightly with increasing age [7].

Fortunately, almost 80% of HPV infections resolve spontaneously within 1–2 years [8]. However, accumulating evidence suggests that HPV infection is more likely to persist during pregnancy [9] and regress after delivery [10–13]. Indeed, the increase of steroid hormones during pregnancy could modify the maternal immune system and contribute to the “tolerance” of the fetus but decrease the ability to clear infections, including HPV [14].

Maternal infections, inflammation [15], and changes in vaginal bacterial microbiota [16] have been recognized as an underlying cause of major adverse pregnancy outcomes (APO) such as miscarriage [17], spontaneous preterm birth [18], and pregnancy-related hypertension disorders [19]. In particular, a recent large multicenter study carried out across eight clinical sites in the USA reported that infections/inflammation are involved in 38% of cases of spontaneous premature delivery [18]. This proportion could even be underestimated, as some of pathogens are under detected because of the lack of sensitivity of conventional detection techniques and difficulties in taking informative samples when the infection is intrauterine [15]. In addition, some genital infections remain asymptomatic and, as such, are not diagnosed [20].

Especially, HPV infections possess those peculiarities in terms of long-term latency, episodic detectability, and genital as well as intrauterine localization. Indeed, genital HPV infection may have a long-term clinical latency, which either reflects the viral persistence (defined as two or more HPV-positive tests over a certain period [21]) or viral latency (HPV is not cleared but remains undetectable by conventional molecular tests and can eventually reactivate) [22]. Clinical latency may encompass episodic states of viral clearance and recurrence, which can lead to misinterpretation of HPV infection status based only on one or few HPV tests [23]. Furthermore, HPV has been localized in amniotic fluid [24], in placental trophoblastic cells [25, 26] and umbilical cord blood [26, 27]. Sometimes, intrauterine infection does not necessary coincide with cervical infection [25].

In vitro studies [28–30] and animal models [31–33] demonstrated biological plausibility of detrimental effect of HPV on pregnancy outcomes. These experimental studies have shown that HPV can replicate in trophoblasts [30] leading to (1) inhibition of blastocyst formation [32]; (2) failed or suboptimal endometrial implantation of trophoblastic cells [29, 31]; and (3) apoptosis of embryonic cells [33]. Besides these direct effects on placenta cells, it has been hypothesized that HPV-trophoblast interaction may trigger immune hypersensitivity to bacteria leading to pregnancy complications, such as preeclampsia or preterm labor [34].

Along with these experimental evidences, observational studies have investigated the effect of HPV on APO, but their results are equivocal. Some authors have concluded to the association between HPV and APO [35–39], whereas others have not found any association [40–43] or have found that HPV is not an independent risk factor of APO but could potentiate the harmful effect of other genital infections on pregnancy [42, 44].

HPV infections can be a source of anxiety for pregnant women and their family. Indeed, health professionals have the task to answer questions and reassure HPV-positive women attending antenatal clinics on the risk of APO related to HPV infection. With this kind of inconsistent data, systematic reviews are recommended for identification, appraisal, and synthesis of evidence in order to inform policy makers and clinicians [45].

Two reviews [46, 47] both published in 2014 have focused on the question of HPV and pregnancy complications. Bonde et al. reviewed the association between HPV infection and infertility, APO, and the risk of vertical transmission of HPV [46]. This review did not follow any systematic approach to document and synthesize the existing literature. After a narrative assessment of the literature, the authors concluded that more research was necessary to reach firm conclusions on the association between pregnancy outcomes and HPV [46]. Conversely, Huang et al. conducted a meta-analysis and found a twofold increased risk of preterm birth in pregnant women infected by HPV (pooled OR 2.12, 95% CI 1.51–2.98) [47]. Although several subgroup analyses were conducted to explore the substantial heterogeneity (*I* ^*2*^ of 61%), this review still contained several limitations such as important exposure measurement bias. Indeed, in three of eight included studies, HPV detection was prior to pregnancy [39, 48] or postnatal [37]. Given the high clearance rate of HPV [8] and considering that pregnancy represents the risk period, measuring exposure out of the pregnancy period might lead to exposure measurement bias. However, the effect of this bias on the pooled estimate has not been assessed.

In addition to the time point of HPV detection, other factors, such as exposure measurement methods (cytology vs HPV test) and characteristics of participants (e.g., other genital infections) acting as confounding factors, could explain the substantial heterogeneity in this review and could have led to a biased estimate. Also, the assessment of confidence in cumulative evidence was limited to publication bias analysis. Finally, there could be residual confounding that may explain the large magnitude effect reported by this meta-analysis.

Clinical trials have shown HPV vaccines to be highly efficacious in preventing infections with the HPV types targeted by the vaccines [49]. There is a growing literature on the promising benefits of HPV vaccination to prevent HPV-related APO [50, 51]. In fact, widespread implementation of HPV vaccination programs is expected to reduce HPV prevalence and diseases related to HPV [49, 52]. Especially, HPV vaccination is expected to reduce incidence of cervical cancer and pre cancers, which will reduce the need for cervical excision and therefore the number of preterm birth [46, 50, 53]. However, there is insufficient evidence on the potential harmful effect of HPV on pregnancy outcomes to support this potential benefit of HPV vaccine on pregnancy outcomes.

Given the rarity and limited quality of evidence of previous reviews, there is a need to conduct a systematic review using a clear and reproducible method taking into account limitations of previous reviews (exposure window, measurement of exposure, and uncontrolled confounding). We will follow recommended guidelines to conduct an extensive and systematic search, critically appraise and summarize the search results and thoroughly assess the quality of the overall evidence. We will also use sensitivity analyses to assess the impact of potential limitations. Thus, we will provide a better understanding of contradictory previous studies and recent literature on the effect of HPV infection on pregnancy outcomes. Ultimately, the current review could shed light on the level of confidence in overall evidence on the association between HPV infection and APO.

## Objectives

Our main objective is to assess whether HPV infection in pregnant women is associated with APO.

## Methods

This protocol will follow the preferred reporting items for systematic review and meta-analysis protocols (PRISMA-P 2015) [54]. In interest of transparency and completeness, a completed PRISMA-P 2015 checklist is provided [see Additional file 1].

### Eligibility criteria

The search strategy has been developed on the basis of the PICOS framework: *P*opulation, *I*ntervention (or Exposure), *C*omparator, *O*utcomes and *S*tudy design.

#### Types of studies

We will include all types of observational studies: cohort (retrospective and prospective), case-control, and cross-sectional studies. We will also consider data from placebo groups of HPV vaccination randomized controlled trials, which might have documented HPV-associated APO.

#### Population of interest

We will include studies investigating APO in women with and without HPV infection.

#### Exposure

The exposure of interest is the presence compared to the absence of HPV infection in pregnant women. We will consider HPV infection in all genital sites (vulva, vagina, or cervix), in placenta, or other products of conception (POC). The presence or absence of HPV infection will need to have been proven by a sensitive molecular method. We will include studies reporting either on overall HPV infection or on HPV type-specific infection.

Findings from in vitro studies and animal models proved that HPV replication in the trophoblasts is the principal cause leading to various HPV-related APO [28–34]. Practically, it is difficult to distinguish the exact exposure time window, which reflects the period during which the exposure (HPV infection) is having its effects relevant to the outcomes of interest (APO) [55]. We assume that the pregnancy period represents the exposure time window, which is the time period during which HPV infection exerts its deleterious effect on pregnancy outcome. Thus, we will include studies that reported HPV infection during pregnancy. However, the median duration of HPV infection has been estimated at approximately 12 months in a recent multi-country study of healthy women aged over 25 years [8]. Consequently, HPV infection detected prior to pregnancy may persist during pregnancy and even after. Therefore, we will also consider studies that reported HPV detection within 12 months prior to pregnancy and after delivery. The effect of these exposure window assumptions and inclusion of studies that used cytology as exposure measurement will be assessed in sensitivity analyses.

#### Comparators

The comparison groups will include pregnant women who are not infected by HPV, matched or not for known possible confounders (age, smoking, alcohol, or other genital infections). For the studies on HPV infection in POC, comparators of concern will be POC in women without HPV infection.

#### Outcomes and prioritization

Prioritization and definitions of the outcomes of interest, with sources of definitions in brackets, are described below:

##### Primary outcomes

For the purpose of this review, we will primarily focus on APO for which there is a plausible biological HPV role suggested by in vitro studies. Therefore, our main outcomes of interest will include the following:

1. Pregnancy loss (or spontaneous abortion or miscarriage), defined as a nonviable, intrauterine pregnancy with either an empty gestational sac, or a gestational sac containing an embryo or fetus without fetal heart activity within the first 12 weeks of gestation (early pregnancy loss) or within the first 20 weeks (late or second trimester pregnancy loss) (ACOG: American College of Obstetricians and Gynecologists) [56]
2. Preterm birth (PTB), defined as birth between 20^0/7^ and 36^6/7^ weeks of gestation (ACOG) [57]. Studies without distinction between spontaneous and elective preterm birth will be included. Where feasible, this aspect will be assessed in subgroup or sensitivity analysis.

##### Secondary outcomes

Secondary outcomes consist of APO that have been associated with maternal genital viral infections in general [58] and with HPV in particular [34] or have been linked to primary outcomes [59]. Thus, we will include APO that may be associated with PTB (such as pregnancy-induced hypertensive disorders (PIHD) [59, 60]) or that may be on the causal pathway of PTB (such as preterm pre-labor rupture of membranes (PPROM) [61]. In addition, secondary outcomes will include low birth weight (LBW) as a proxy of PTB [59] and intrauterine growth restriction (IUGR) as a constituent of LBW [59]. We will include studies that reported on birth defects as almost 50% of early miscarriages are due to chromosomal abnormalities [56]. Likewise, we will consider stillbirths, which are related to infection in general [62] and to most of previous APO such as PIHD, IUGR, and PPROM [63].

The following APO will be considered as secondary outcomes of interest:

1. LBW, defined as a weight of less than 2500 g irrespective of the gestational age (WHO-ICD-10: World Health Organization-International statistical Classification of Diseases-tenth revision) [64].
2. PIHD, including preeclampsia/eclampsia and gestational hypertension. Preeclampsia is a syndrome defined by hypertension (blood pressure of 140 mmHg systolic or higher or 90 mmHg diastolic or higher that occurs after 20 weeks of gestation in a woman with previously normal blood pressure) and proteinuria (urinary excretion of 0.3 g protein or higher in a 24-h urine specimen). Eclampsia is defined as the presence of new-onset grand mal seizures in a woman with preeclampsia. The term gestational hypertension refers to a woman who develops an elevated blood pressure without proteinuria after 20 weeks of gestation and blood pressure levels return to normal postpartum (ACOG) [65].
3. PPROM, defined as membrane rupture before labor and before 37 weeks of gestation (ACOG) [66]
4. IUGR, defined as a fetus with an estimated fetal weight <10th percentile at birth (SOGC: Society of Obstetricians and Gynecologists of Canada) [67].
5. Birth defects. For practical reasons, we will focus on birth defects that may be noticed at birth and for which there are screening and diagnostic tests during pregnancy [68].
6. Stillbirth, defined as fetal death that occurs during pregnancy at 20 weeks of gestation or greater [63].

#### Settings

There will be no period or geographical restriction.

#### Language

There will be no language restriction provided that there is an English or French abstract. Excluded English or French relevant titles without English or French abstract will be provided as an appendix.

### Information sources

Our systematic search will target three information sources. We will initially target MEDLINE, EMBASE, and EBM Reviews databases (Cochrane Database of Systematic Reviews, American College of Physicians- ACP- Journal Club, Database of Abstracts of Reviews of Effects, Cochrane Central Register of Controlled Trials, Cochrane Methodology Register, Health Technology Assessment and National Health Service –NHS- Economic Evaluation Database). We will search MEDLINE (PubMed and Ovid interfaces) from 1946, EMBASE from 1981, and EBM (Ovid interface) from 1991 onwards.

To ensure a complete search coverage, we will hand-search reference lists of included titles and of any previous review related to our research question. Moreover, we will search for additional relevant titles by screening citations of included articles using Google Scholar or Web of Science.

Finally, in order to identify unpublished studies, we will search grey literature through Google Scholar and Web of Science. Especially, we will search relevant conference proceedings published on websites of international conferences on HPV, such as IPV (International Papillomavirus) and EUROGIN (European Research Organization on Genital Infection and Neoplasia) conferences.

### Search strategy

We will combine three systematic search strategies to identify relevant literature. Initially, for each of electronic databases, a specific search strategy was developed. We have first developed a search strategy for MEDLINE based on Medical Subject Headings (MeSH) and free text words. Our research team developed the terms for MEDLINE on the basis of the eligibility criteria. The search strategy was developed by the principal author (JN) and a health sciences librarian with expertise in systematic literature searching. We combined terms (or exploded terms where possible) using Boolean operators. Terms developed in MEDLINE were adapted for correct use in the EMBASE and EBM Reviews databases. Preliminary search strategies piloted for PubMed, EMBASE, and EBM Reviews databases are provided [see Additional file 2].

The second search strategy will target grey literature to identify additional unpublished articles in journals indexed in electronic databases. To maximize identification of more relevant studies, we will combine three complementary grey literature online sources, namely Google Scholar, Web of Science conference proceedings citation index, and International conferences specific websites. Thus, we will search Google Scholar using the following free text words: “pregnancy outcomes” and “papillomavirus”. We will retain relevant unpublished titles, which will be screened according to the eligibility criteria. The same search terms used for MEDLINE will be adapted for Web of Science. We will apply filters within Web of Science search strategy to restrict results to conference proceedings. In addition, we will hand-search for relevant conference proceedings of not formally published studies through online abstract books of IPV and Eurogin conferences.

The third search strategy will target additional relevant titles by searching citations of included articles and of any previous review. We will screen reference lists of included studies and previous reviews to find relevant cited articles. We will use Google Scholar (or Web of Science) to find citing publications of included articles. Citing and cited articles will be screened using the same inclusion and exclusion criteria as for the two previous search strategies.

### Study records

#### Data management

All titles identified from electronic databases (MEDLINE, EMBASE, and EBM Reviews databases) will be uploaded and combined into an EndNote file (EndNote X7.3). Titles retrieved from Grey literature will be manually entered into EndNote file or uploaded individually from Google Scholar. Using EndNote’s auto-deduplication function, we will first automatically find and remove duplicate publications. To avoid any residual duplicate, we will sort the remaining records by author’s names and later by titles. For the sake of clarity, all duplicates will be kept into duplicate EndNote subfolder. After duplicates are removed, all remaining records will be saved into an EndNote subfolder.

#### Selection process

Two authors JN and NZ will independently and in duplicate screen the remaining records in two phases.

At the first phase (*screening*), a standardized checklist created on the basis of eligibility criteria will be used to screen titles and abstracts [see Additional file 3]. The screening phase will differentiate potentially eligible from non-eligible studies. Thus, study’s title or abstract that clearly does not pertain to any of PICOS criteria will be excluded. Studies that cannot be clearly excluded on the basis of title or abstract (uncertain and eligible) will be retained for further assessment.

At the second phase (*eligibility*), full text reports of all potentially eligible records will be retrieved and independently analyzed by the same two reviewers. A standardized form with explicit and detailed eligibility criteria on the basis of PICOS will be used to screen full text reports [see Additional file 4].

With the aim of reducing errors, a pilot test of the standardized tools will be conducted prior to the complete selection process. At each selection step, a random sample of 10% of the records will be used to test screening and eligibility tools. Disagreements will be resolved by discussion between the two reviewers. Any persisting discrepancy will be discussed with the senior researchers (HT and MHM).

In the interest of transparency, a list of excluded studies after full text assessment and detailed reasons of exclusion will be provided in review additional files. A PRISMA flow diagram will summarize the entire selection process. For each selection step, the flow diagram will indicate the number of retained and excluded records with a short description of the reasons for exclusion.

#### Data collection process

Data will be extracted from selected studies using a standardized form adapted from the Effective Practice and Organization of Care (EPOC) data collection form [69] (Additional file 4). To minimize errors in data collection, a pilot test of standardized form will be conducted on a random sample of 10% of included studies. The form will be revised as needed to insure that relevant information is collected. The two first reviewers will independently and in duplicate extract data from full text reports of included studies. Discrepancies will be resolved through consensus and if necessary by discussion with a third senior author. In the absence of important information (such as the number of APO and total sample size), we plan to contact (with a maximum of three email attempts) the primary author of included studies to obtain missing data.

### Data items

Extracted data will include, but not be limited to, study characteristics (first author name, country, and year of publication), methods (aim of study, design, unit of observation, definition of APO), participants’ characteristics (source population description, inclusion and exclusion criteria, methods of recruitment, sample size, average age, HPV detection procedures, time of HPV detection), and number of each APO within HPV-exposed and HPV-unexposed groups. HPV genotypes will be extracted when possible. Some studies may include a composite measure of APO. We will extract, if possible, frequency of each APO from the composite outcome. If the information on each component of composite outcome is not available, we plan to contact the primary author to obtain missing data (with a maximum of three email attempts); otherwise, we will report the composite outcome with its definition provided in individual studies.

### Risk of bias in individual studies

We will use the Effective Public Health Practice Project (EPHPP) Quality Assessment Tool for Quantitative Studies to evaluate methodological quality and risk of bias of included studies. The EPHPP is a standardized tool relevant to evaluate quality of quantitative observational studies [70]. This quality assessment tool mainly encompasses the Strengthening the Reporting of Observational Studies in Epidemiology (STROBE) checklist. The EPHPP has acceptable content and construct validity [71] as well as excellent inter-rater reliability compared to the Cochrane Collaboration Risk of Bias Tool (CCRBT) [70]. The EPHPP tool evaluates six domains of study validity: selection bias, study design, confounders, blinding, data collection method, and withdrawals/dropouts [72]. Each of six domains can be rated as strong, moderate, or weak. Based on individual domain ratings, the quality of each included study will be globally scored as either (1) strong (no weak individual domain rating); (2) moderate (one weak individual domain rating), or (3) weak (two or more weak individual domains ratings) [72]. This bias and quality assessment will be independently and in duplicate carried out by the two first reviewers. We will compute agreement in individual domain ratings between reviewers for each included primary study based on the Cohen kappa coefficient. Discrepancies and study component ratings with kappa values below 0.80 will be resolved through discussion. Unresolved disagreement will be discussed with a third author. Differences among studies revealed by quality assessment of primary studies will guide, where appropriate, subsequent subgroup, or sensitivity analyses [73].

### Data synthesis

The purpose of our data synthesis will be twofold: (1) a narrative synthesis through which we will summarize and discuss findings of included studies and (2) a statistical analysis whereby we will investigate relationship between APO and HPV infection.

#### Narrative synthesis

We will narratively synthesize included studies whether meta-analysis is appropriate or not. For the purpose of transparency and reproducibility, we will adopt, where applicable, the narrative synthesis guidance proposed by Popay et al. [74]. The guidance describes a framework of four elements on which the narrative synthesis may be based: (1) developing a theory of how the intervention works, why, and for whom, (2) developing a preliminary synthesis of findings of included studies, (3) exploring relationships within and between studies, and (4) assessing the robustness of the synthesis. It is not required to proceed linearly or to apply all four elements in narrative synthesis. However, one chooses the elements to be employed depending on the nature of research question being reviewed [75]. According to the objective of this review, we plan to use only the 2nd and 3rd elements of this guideline.

We will initially present findings first by primary outcomes and then by secondary outcomes. We will use tabular summary to synthesize individual studies characteristics and results (direction and magnitude of effects). In addition, we will explore factors, which might explain differences in direction and size of effect within the included studies [45, 74]. With particular consideration to potential confounders, we will assess results in light of participant’s characteristics and exposure measurement differences. Without computing a pooled estimate, we will visually assess the dispersion of effect estimates from primary studies using forest plots. We will finally conclude the narrative synthesis by an assessment of publication bias and strength of overall evidence.

#### Statistical analysis

We anticipate that there will be much variability between included studies because of clinical diversity (variability in the characteristics of participants, exposure measurement and outcomes) and methodological diversity (variability in study design and risk of bias). Thus, we will conduct a meta-analysis using Der Simonian-Laird random-effects model for each primary and secondary outcome [76]. We assume that included studies will report dichotomous outcomes data from HPV-exposed and HPV-non-exposed pregnant women or products of conception. We will then, for each study, compute a weighted measure of association as relative risk (RR) estimated with the comparison of exposed women to unexposed women. However, it is worth mentioning that focusing data analysis solely on the average measure of random effects may be misleading [77]. We will therefore put emphasis on the analysis of the dispersion of the measures of association of individual studies rather than focusing on summary estimates yielded by meta-analyses. This analysis will be focused, but not be limited, to the assessment of the extent of inconsistency, the amount of dispersion, and the causes of heterogeneity. Particularly, heterogeneity assessment will include investigation of the effect of the major limitations of previous reviews: exposure window, measurement of exposure, and uncontrolled confounding. All statistical analyses will be conducted using STATA 14 (StataCorp. 2015. Statistical Software: Release 14. College Station, TX: StataCorp LP.).

1. *Assessment of the extent of inconsistency*
  We will use forest plots to represent the dispersion of observed RRs. Along with the forest plot, we will compute *I* ^*2*^ statistic (with its 95% CI) to formally quantify the proportion of variance in observed RRs that reflects the true heterogeneity between studies rather than chance [45]. According to criteria of Higgins et al., we will consider *I* ^*2*^ of more than 50% as substantial heterogeneity [45].
2. *Assessment of the amount of dispersion*
  In addition to *I* ^*2*^, we will report *T* ^*2*^ statistic as the absolute amount of dispersion of true effects, especially in case of meta-analyses with few studies [77]. We will also compute the 95% prediction interval to estimate the range within which a hypothetical new true measure of association is expected to be found in 95 of 100 cases [77]
3. *Investigating the causes of heterogeneity and limitations of previous reviews:*
  - *Subgroup analyses and meta-regression*
    We will investigate through subgroup analyses how clinical and methodological variability influence the pooled measure of association. Although both clinical and methodological diversity lead to statistical heterogeneity [45], we assume that clinical factors may vary more across included studies than methodological factors. Thus, clinical diversity could be more prone to affect clinical meaningful of an average summary estimate and heterogeneity than methodological diversity. Subgroup analyses will therefore be focused on the assessment of the effect of key characteristics of participants identified from literature, which may be associated with the primary outcomes of interest and uncontrolled in previous reviews. Thus, we will conduct subgroup analyses based, but not limited to, maternal age, parity, race, smoking, drug use, genital infections, and prior APO. We will also group and compare studies that have matched/controlled and not matched/not controlled data on some of these key variables (age, parity, and smoking). Where appropriate, we will conduct a meta-regression on the basis of study average or proportion values of key participant’s characteristics.
  - *Sensitivity analyses*
    We will assess robustness of our findings by restricting the analysis to a subset of studies with high or moderate quality based on EPHPP score. Sensitivity analysis will be undertaken to take into account quality relative to confounding bias by stratification of most adjusted and least adjusted estimates from each study. Similarly, we plan to assess the effect of time point detection of HPV by restricting analysis to different alternative exposure windows (12 months prior to pregnancy, pregnancy period, or 12 months postpartum). Finally, we will investigate the extent to which inclusion of studies that used cytology as exposure measurement affects the results.

### Meta-biases assessment

If there are at least 10 studies for each outcome, we will use funnel plot to explore the potential of publication bias and small studies effect [45]. Additionally, Egger’s test for funnel plot asymmetry will be conducted [45]. However, in case of less than 10 studies, we will assess qualitatively the small study effect. For this purpose, we will perform a cumulative meta-analysis sorting studies from largest to smallest study to examine the effect of study size on the pooled estimate [78]. We will assess selective reporting within included studies by comparing outcomes reported in methods and results sections [79].

### Confidence in cumulative evidence

We will use the Grading of Recommendations Assessment, Development and Evaluation (GRADE) approach to assess and grade the confidence of evidence for each outcome across included studies [80]. Quality rating of overall evidence will be downgraded according to five factors: (1) limitations in the design (risk of bias across studies); (2) indirectness (exposure or outcomes are different from those defined in inclusion criteria of this protocol); (3) unexplained heterogeneity (after subgroup analyses); (4) imprecision of effect estimates (few participants or few events); and (5) risk of publication bias [45] [80]. In addition and where appropriate, quality rating will be upgraded according to the following three factors: (1) a large magnitude of effect, (2) a dose response gradient (e.g., the more types of HPV are identified, the high is the risk of pregnancy outcome), and (3) plausible residual confounding that would reduce a demonstrated effect or suggest a spurious effect when results show no effect [45].We will integrate downgrading and upgrading factors to obtain an overall quality of evidence for each outcome of interest. Overall quality of evidence will be then ranked as high, moderate, low, or very low as specified by GRADE approach [80]. However, in order to be more explicit about overall level of confidence of our findings, we will translate GRADE levels into categories suggested by Young et al. [81] as high (further research is very unlikely to change our confidence in the estimate of effect), moderate (further research is likely to have an important impact on our confidence in the estimate of effect and may change the estimate), low (further research is very likely to have an important impact on our confidence in the estimate of effect and is likely to change the estimate), or very low (very uncertain about the estimate of effect). Finally, key information concerning the quality of evidence of all outcomes will be concisely combined in “summary of findings” table.

## Discussion

This review will rely only on observational studies, which are prone to confounding biases. Moreover, we expect that there will be much heterogeneity in included studies in terms of study design, exposure measurement, characteristics of participants, and outcomes. Nevertheless, to our knowledge, this will be the first review that will use a validated systematic and transparent methodological process to appraise and summarize the existing literature on HPV-associated pregnancy outcomes. Thus, whatever conclusion our study reaches (harmful effect, absence of effect, or uncertain effect), we are confident that this review will provide rigorous and comprehensive high-quality evidence related to HPV-associated pregnancy outcomes. Indeed, we expect that our findings will ultimately fall into one of the following three scenarios. Firstly, if our findings are in favor of a harmful effect of HPV infection on pregnancy outcomes, this might explain a part of APO so far classified as idiopathic. Therefore, the fact that HPV infection might explain some APO could strengthen primary prevention through vaccination. Secondarily, in case of absence of relationship, this might dispel anxiety and reassure HPV-infected pregnant women and health professionals. Thirdly, if ever we end up being uncertain of the effect, we will provide an overview of gaps in current knowledge and propose further research directions on HPV-pregnancy outcome relationship.

## Dissemination of findings

We will report this review in accordance to the Meta-analysis of Observational Studies in Epidemiology (MOOSE) guidelines [82] and the Preferred Reporting Items for Systematic Reviews and Meta-analyses (PRISMA) [83]. This systematic review will be part of JN’s PhD research thesis co-supervised by HT and MHM. This systematic review will also be submitted for publication in a relevant peer-reviewed journal, and findings will be presented in scientific conferences.

## Additional files

Additional file 1: PRISMA-P checklist (DOCX 26 kb)
Additional file 2: Preliminary search strategy (DOCX 17 kb)
Additional file 3: Screening titles and abstracts (DOCX 13 kb)
Additional file 4: Data extraction form (DOCX 26 kb)

## Abbreviations

ACOG: American College of Obstetricians and Gynecologists
APO: Adverse pregnancy outcomes
CCRBT: Cochrane Collaboration Risk of Bias Tool
CIHR: Canadian Institutes of Health Research
EPHPP: Effective Public Health Practice Project Quality Assessment Tool for Quantitative Studies
EPOC: Effective Practice and Organization of Care
EUROGIN: European Research Organization on Genital Infection and Neoplasia conference
FRQ-S: Fonds de la Recherche du Québec en Santé
GRADE: Grading of Recommendations Assessment, Development and Evaluation
HPV: Human papillomavirus
IPV: International Papillomavirus conference
IUGR: Intrauterine growth restriction
LBW: Low birth weight
MeSH: Medical Subject Headings
MOOSE: Meta-analysis of Observational Studies in Epidemiology guidelines
PICOS: Population, Intervention-Exposure, Comparator, Outcomes and Study design
PIHD: Pregnancy-induced hypertensive disorders
POC: Products of conception
PPROM: Preterm pre-labor rupture of membranes
PRISMA: Preferred Reporting Items for Systematic Reviews and Meta-analyses
PRISMA-P: Preferred Reporting Items for Systematic review and Meta-Analysis Protocols
PTB: Preterm birth
QTNPR: Quebec Training Network in Perinatal Research
SOGC: Society of Obstetricians and Gynecologists of Canada
STROBE: Strengthening the Reporting of Observational Studies in Epidemiology
WHO-ICD-10: World Health Organization-International statistical Classification of Diseases-tenth revision
