# Supplementary material for: The association between adverse pregnancy outcomes and maternal human papillomavirus infection: a systematic review protocol
Source: Syst Rev. 2017 Mar 11;6:53. doi: 10.1186/s13643-017-0443-5 (PMC5346269; doi:10.1186/s13643-017-0443-5)
Supplement: Additional file 2: — Preliminary search strategy (DOCX 17 kb) [file 13643_2017_443_MOESM2_ESM.docx]

**Additional file 2: Preliminary Search strategy**

**PubMed**

1. (((((Pregnant Women[mh] OR Pregnancy[mh] OR Pregnan*[tiab] OR conception[tiab] OR gravid*[tiab] OR gestation*[tiab] OR Childbearing[tiab] OR "Child bearing"[tiab] OR parturient[tiab] OR parturition[tiab] OR expectant[tiab] OR Placenta*[tiab] OR Pregnan*[OT] OR conception[OT] OR gravid*[OT] OR gestation*[OT] OR Childbearing[OT] OR "Child bearing"[OT] OR parturient[OT] OR parturition[OT] OR expectant[OT] OR Placenta*[OT])
2. (Infant, Premature[mh] OR Infant, Low Birth Weight[mh] OR preterm[tiab] OR premature[tiab] OR prematurity[tiab] OR low birthweight[tiab] OR low-birth weight[tiab] OR LBW[tiab] OR VLBW[tiab] OR ELBW[tiab] OR small for gestational age[tiab] OR Small for Gestation Age[tiab] OR smallness for gestational age[tiab] OR SGA[tiab] OR hypertensive disorder*[tiab] OR Hypertension[tiab] OR preterm[OT] OR premature[OT] OR prematurity[OT] OR low birthweight[OT] OR low-birth weight[OT] OR LBW[OT] OR VLBW[OT] OR ELBW[OT] OR small for gestational age[OT] OR Small for Gestation Age[OT] OR "smallness for gestational age"[OT] OR SGA[OT] OR hypertensive disorder*[OT] OR Hypertension[OT])) OR (Pregnancy Outcome[mh] OR Pregnancy Complications[mh] OR pregnancy outcome*[tiab] OR pregnancy complication*[tiab] OR pregnancy adverse outcome*[tiab] OR pregnancy loss*[tiab] OR pregnancy toxemia*[tiab] OR pregnancy toxaemia*[tiab] OR Gestational hypertension[tiab] OR Abortion*[tiab] OR Miscarriage*[tiab] OR Stillbirth*[tiab] OR Still-birth*[tiab] OR Perinatal outcome*[tiab] OR Perinatal complication*[tiab] OR Perinatal adverse outcome*[tiab] OR Obstetric outcome*[tiab] OR Obstetric complication*[tiab] OR Obstetric adverse outcome*[tiab] OR Preeclampsia*[tiab] OR Eclampsia*[tiab] OR Maternal outcome*[tiab] OR Maternal complication*[tiab] OR Maternal adverse outcome*[tiab] OR "Reproductive outcome"[tiab] OR "Reproductive adverse outcome"[tiab] OR "Reproductive adverse outcomes"[tiab] OR "Reproductive outcomes"[tiab] OR HELLP syndrome[tiab] OR fetal growth restriction[tiab] OR fetal growth retardation[tiab] OR foetal growth restriction[tiab] OR foetal growth retardation[tiab] OR premature rupture of membrane*[tiab] OR "premature rupture of fetal membrane"[tiab] OR "premature rupture of foetal membrane"[tiab] OR Poor Fetal Growth[tiab] OR Poor Foetal Growth[tiab] OR pregnancy outcome*[OT] OR pregnancy complication*[OT] OR pregnancy adverse outcome*[OT] OR pregnancy loss*[OT] OR pregnancy toxemia*[OT] OR pregnancy toxaemia*[OT] OR Gestational hypertension[OT] OR Abortion*[OT] OR Miscarriage*[OT] OR Stillbirth*[OT] OR Still-birth*[OT] OR Perinatal outcome*[OT] OR Perinatal complication*[OT] OR Obstetric outcome*[OT] OR Obstetric complication*[OT] OR Obstetric adverse outcome*[OT] OR "Reproductive adverse outcome"[OT] OR "Reproductive outcome"[OT] OR "Reproductive adverse outcomes"[OT] OR "Reproductive outcomes"[OT] OR Preeclampsia*[OT] OR Eclampsia*[OT] OR Maternal outcome*[OT] OR Maternal complication*[OT] OR HELLP syndrome[OT] OR fetal growth restriction[OT] OR fetal growth retardation[OT] OR foetal growth restriction[OT] OR "foetal growth retardation"[OT] OR premature rupture of membrane*[OT] OR "premature rupture of fetal membrane"[OT] OR "premature rupture of foetal membrane"[OT] OR "Poor Fetal Growth"[OT] OR "Poor Foetal Growth"[OT]))
3. (Papilloma[mh] OR Papillomaviridae[mh:noexp] OR Alphapapillomavirus[mh] OR Papillomavirus Infections[Mh] OR Papilloma*[tiab] OR Alphapapillomavir*[tiab] OR HPV[tiab] OR Genital Wart*[tiab] OR Venereal Wart*[tiab] OR Anogenital Wart*[tiab] OR Ano-genital Wart*[tiab] OR Anal Wart*[tiab] OR Condylomata Acuminat*[tiab] OR Papilloma*[OT] OR Alphapapillomavir*[OT] OR HPV[OT] OR Genital Wart*[OT] OR Anogenital Wart*[OT] OR Anal Wart*[OT] OR Condylomata Acuminat*[OT]))
4. (1 and 2 and 3) NOT ((animals[mh] NOT humans[mh]))) =971 (as of April 13, 2016)

**Medline Ovid**

1 Exp Papilloma/ OR Papillomaviridae/ OR Exp Alphapapillomavirus/ OR Exp Papillomavirus Infections/ OR (Papilloma* OR Alphapapillomavir* OR HPV OR Genital Wart* OR Venereal Wart* OR Anogenital Wart* OR Ano-genital Wart* OR Anal Wart* OR Condylomata Acuminat*).ti,ab,kf

2 Exp Pregnancy Outcome/ OR Exp Pregnancy Complications/ OR (pregnancy outcome* OR pregnancy complication* OR pregnancy adverse outcome* OR pregnancy loss* OR pregnancy toxemia* OR pregnancy toxaemia* OR Gestational hypertension OR Abortion* OR Miscarriage* OR Stillbirth* OR Still-birth* OR Perinatal outcome* OR Perinatal complication* OR Perinatal adverse outcome* OR Obstetric outcome* OR Obstetric complication* OR Obstetric adverse outcome* OR Preeclampsia* OR Eclampsia* OR Maternal outcome* OR Maternal complication* OR Maternal adverse outcome* OR Reproductive adverse outcome* OR Reproductive outcome* OR HELLP syndrome OR fetal growth restriction OR fetal growth retardation OR foetal growth restriction OR foetal growth retardation OR premature rupture of membrane* OR "premature rupture of fetal membrane" OR "premature rupture of foetal membrane" OR Poor Fetal Growth OR Poor Foetal Growth).ti,ab,kf

3 Pregnant Women/ OR Exp Pregnancy/ OR (Pregnan* OR conception OR gravid* OR gestation* OR Childbearing OR "Child bearing" OR parturient OR parturition OR expectant OR Placenta*).ti,ab,kf

4 Exp Infant, Premature/ OR Exp Infant, Low Birth Weight/ OR (preterm OR premature OR prematurity OR low birthweight OR low-birth weight OR LBW OR VLBW OR ELBW OR small for gestational age OR Small for Gestation Age OR smallness for gestational age OR SGA OR hypertensive disorder* OR Hypertension).ti,ab,kf

1. exp animals/ not exp humans/
2. (1 and (2 or (3 and 4))) not 5=939 (as of April 13, 2016)

**EBM Reviews**

1 Exp Papilloma/ OR Papillomaviridae/ OR Exp Alphapapillomavirus/ OR Exp Papillomavirus Infections/ OR (Papilloma* OR Alphapapillomavir* OR HPV OR Genital Wart* OR Venereal Wart* OR Anogenital Wart* OR Ano-genital Wart* OR Anal Wart* OR Condylomata Acuminat*).ti,ab,kf

2 Exp Pregnancy Outcome/ OR Exp Pregnancy Complications/ OR (pregnancy outcome* OR pregnancy complication* OR pregnancy adverse outcome* OR pregnancy loss* OR pregnancy toxemia* OR pregnancy toxaemia* OR Gestational hypertension OR Abortion* OR Miscarriage* OR Stillbirth* OR Still-birth* OR Perinatal outcome* OR Perinatal complication* OR Perinatal adverse outcome* OR Obstetric outcome* OR Obstetric complication* OR Obstetric adverse outcome* OR Preeclampsia* OR Eclampsia* OR Maternal outcome* OR Maternal complication* OR Maternal adverse outcome* OR Reproductive adverse outcome* OR Reproductive outcome* OR HELLP syndrome OR fetal growth restriction OR fetal growth retardation OR foetal growth restriction OR foetal growth retardation OR premature rupture of membrane* OR "premature rupture of fetal membrane" OR "premature rupture of foetal membrane" OR Poor Fetal Growth OR Poor Foetal Growth).ti,ab,kf

3 Pregnant Women/ OR Exp Pregnancy/ OR (Pregnan* OR conception OR gravid* OR gestation* OR Childbearing OR "Child bearing" OR parturient OR parturition OR expectant OR Placenta*).ti,ab,kf

4 Exp Infant, Premature/ OR Exp Infant, Low Birth Weight/ OR (preterm OR premature OR prematurity OR low birthweight OR low-birth weight OR LBW OR VLBW OR ELBW OR small for gestational age OR Small for Gestation Age OR smallness for gestational age OR SGA OR hypertensive disorder* OR Hypertension).ti,ab,kf

5 exp animals/ not exp humans/

6 (1 and (2 or (3 and 4))) not 5 =16 (on 13/04/2016)

**Embase**

1 Papilloma/ OR papillomaviridae/ or exp alphapapillomavirus/ or exp wart virus/ or exp papillomavirus infection/ OR (Papilloma* OR Alphapapillomavir* OR HPV OR Genital Wart* OR Venereal Wart* OR Anogenital Wart* OR Ano-genital Wart* OR Anal Wart* OR Condylomata Acuminat*).ti,ab,kw

2 Pregnancy Outcome/ OR Exp Pregnancy Complication/ OR Exp pregnancy disorder/ OR (pregnancy outcome* OR pregnancy complication* OR pregnancy adverse outcome* OR pregnancy loss* OR pregnancy toxemia* OR pregnancy toxaemia* OR Gestational hypertension OR Abortion* OR Miscarriage* OR Stillbirth* OR Still-birth* OR Perinatal outcome* OR Perinatal complication* OR Perinatal adverse outcome* OR Obstetric outcome* OR Obstetric complication* OR Obstetric adverse outcome* OR Preeclampsia* OR Eclampsia* OR Maternal outcome* OR Maternal complication* OR Maternal adverse outcome* OR Reproductive adverse outcome* OR Reproductive outcome* OR HELLP syndrome OR fetal growth restriction OR fetal growth retardation OR foetal growth restriction OR foetal growth retardation OR premature rupture of membrane* OR "premature rupture of fetal membrane" OR "premature rupture of foetal membrane" OR Poor Fetal Growth OR Poor Foetal Growth).ti,ab,kw

3 Exp named groups by pregnancy/ OR Exp Pregnancy/ OR (Pregnan* OR conception OR gravid* OR gestation* OR Childbearing OR "Child bearing" OR parturient OR parturition OR expectant OR Placenta*).ti,ab,kw

4 Prematurity/ OR Exp low birth weight/ OR (preterm OR premature OR prematurity OR low birthweight OR low-birth weight OR LBW OR VLBW OR ELBW OR small for gestational age OR Small for Gestation Age OR smallness for gestational age OR SGA OR hypertensive disorder* OR Hypertension).ti,ab,kw

5 exp animal/ not exp human/

6 (1 and (2 or (3 and 4))) not 5 =1019 (as of April 13, 2016)
