# Supplementary material for: The association between adverse pregnancy outcomes and maternal human papillomavirus infection: a systematic review protocol
Source: Syst Rev. 2017 Mar 11;6:53. doi: 10.1186/s13643-017-0443-5 (PMC5346269; doi:10.1186/s13643-017-0443-5)
Supplement: Additional file 3: — Screening titles and abstracts (DOCX 13 kb) [file 13643_2017_443_MOESM3_ESM.docx]

**Additional file 3: Screening titles and abstracts**

1. Does the study include pregnant women and/or products of conception (fetuses, placentas)?

YES____ NO____ UNCLEAR____

1. Does the study report on adverse pregnancy outcomes (see definition of the outcomes of interest below)?

YES____ NO____ UNCLEAR____

1. Does the study report on HPV infection (confirmed by HPV test or cervical dysplasia)?

YES____ NO____ UNCLEAR____

1. Is this a relevant study design (cohort, case-control or cross-sectional)?

YES____ NO____ UNCLEAR____

If you answer NO to any of these questions, the citation will be excluded. All other citations will be included for further screening.
