# Supplementary material for: The association between adverse pregnancy outcomes and maternal human papillomavirus infection: a systematic review protocol
Source: Syst Rev. 2017 Mar 11;6:53. doi: 10.1186/s13643-017-0443-5 (PMC5346269; doi:10.1186/s13643-017-0443-5)
Supplement: Additional file 4: — Data extraction form (DOCX 26 kb) [file 13643_2017_443_MOESM4_ESM.docx]

Additional file 4: Data extraction form

Notes:

- Be consistent in the order and style used to describe each report.
- Record any missing or unclear information so to indicate that the information was not found in the study report(s) but not forgotten.

| Review title |
| --- |
| Human Papilloma Virus infection and adverse pregnancy outcomes: a systematic review |
| Study ID *(surname of first author and year first full report of study was published e.g. Smith 2001)* |
|  |
| Notes |

1. General Information

| 1. Date form completed *(dd/mm/yyyy)* |  |
| --- | --- |
| 1. Name/ID of person extracting data |  |
| 1. Reference citation (e.g. Medline) |  |
| 1. Study author contact details |  |
| 1. Publication type *(e.g. full report, abstract, letter)* |  |
| Notes: | |

1. Study eligibility

| Study Characteristics | Eligibility criteria | Eligibility criteria met? | | | Location in text or source *(pg & ¶/fig/table)* |
| --- | --- | --- | --- | --- | --- |
|  |  | **Yes** | **No** | **Unclear** |  |
| **Study design** | Cohort |  |  |  |  |
|  | Case-control |  |  |  |  |
|  | Cross-sectional |  |  |  |  |
| **Participants** | Pregnant women |  |  |  |  |
|  | Product of conception (fetus, placenta) |  |  |  |  |
| **Exposure** | HPV infection (proved by HPV test) |  |  |  |  |
|  | History of dysplasia |  |  |  |  |
| **Outcomes** | Miscarriages |  |  |  |  |
|  | Preterm birth |  |  |  |  |
|  | Low birth weight |  |  |  |  |
|  | Pregnancy -induced hypertensive disorders  Preeclampsia/eclampsia and gestational hypertension |  |  |  |  |
|  | Intrauterine growth restriction |  |  |  |  |
| INCLUDE | EXCLUDE | | | | |
| **Reason for exclusion** |  | | | | |
| **Notes**: |  | | | | |

**DO NOT PROCEED IF STUDY EXCLUDED FROM REVIEW**

1. Characteristics of included studies

**Population and setting**

|  | | **Description**  ***Include comparative information for each group*** | | **Location in text**  *(pg & ¶/fig/table)* |
| --- | --- | --- | --- | --- |
|  |  | **Exposed** | **Non exposed** |  |
| Population description  *(from which study participants are drawn)* | |  |  |  |
| Setting  *(including clinical setting and social context)* | |  |  |  |
| Inclusion criteria | |  |  |  |
| Exclusion criteria | |  |  |  |
| Method/s of recruitment of participants *(e.g. phone, mail, Prenatal care)* | |  |  |  |
| Total sample size | |  |  |  |
| Age (mean, median and range) | |  |  |  |
| Other genital infections | | YES  NO | YES  NO |  |
| Ethnicity *(Proportion)* | | White  Black  Other | White  Black  Other |  |
| Smoking *(Proportion)* | |  |  |  |
| Alcohol *(Proportion)* | |  |  |  |
| Note: |  | | | |

**Methods**

|  | **Descriptions as stated in report/paper** | **Location in text or source** *(pg & /fig/table)* |
| --- | --- | --- |
| **Aim of study** |  |  |
| **Design** |  |  |
| **Unit of observation** |  |  |
| **Exposure measurement** |  |  |
|  | If HPV test, precise the technique used |  |
| **Start date** |  |  |
| **End date** |  |  |
| **Duration of participation**  *(from recruitment to last follow-up)* |  |  |
| **Notes:** | | |

**Outcomes**

|  | | **Description as stated in report/paper**  **(number with outcome/group sample size)** | | **Location in text** *(pg & ¶/fig/table)* |
| --- | --- | --- | --- | --- |
|  | | Exposed | Non Exposed |  |
| **Primary outcomes** | | Miscarriages      /  Preterm birth      / | Miscarriages      /  Preterm birth      / |  |
| **Secondary outcomes** | | Low birth weight      /  Pregnancy induced hypertensive disorders      /  Intrauterine growth retardation      / | Low birth weight      /  Pregnancy induced hypertensive disorders      /  Intrauterine growth retardation      / |  |
| Note: | *Indicate the definition, if not defined as in this protocol* | | | |
